# Perinatal Outcomes of Monochorionic Twin Pregnancies Conceived Naturally Versus through Assisted Reproductive Techniques

**DOI:** 10.3390/jcm12186097

**Published:** 2023-09-21

**Authors:** Alicia Martínez-Varea, Martha Martínez-Gómez, Blanca Novillo, Josep Domenech, José Morales-Roselló, Vicente Diago-Almela

**Affiliations:** 1 Department of Obstetrics and Gynaecology, La Fe University and Polytechnic Hospital, Avenida Fernando Abril Martorell 106, 46026 Valencia, Spain; marthamartinezgomez96@gmail.com (M.M.-G.); bnalamo@gmail.com (B.N.); cm@comv.es (J.M.-R.); diago_vicalm@gva.es (V.D.-A.); 2 Department of Economics and Social Sciences, Universitat Politècnica de València, Camí de Vera s/n, 46022 Valencia, Spain; jdomenech@upvnet.upv.es; 3 Department of Pediatrics, Obstetrics and Gynecology, University of Valencia, Avenida Blasco Ibáñez 15, 46010 Valencia, Spain

**Keywords:** twin gestation, monochorionic diamniotic twin pregnancy, natural conception, assisted reproductive techniques, perinatal outcome

## Abstract

**Objective**: It has been reported that monochorionic twin pregnancies conceived through assisted reproductive techniques (ART) display a higher risk of second-trimester miscarriage, cesarean delivery, and neonatal death than those conceived naturally. The aim of this study was to compare the perinatal outcomes of monochorionic diamniotic (MCDA) twin pregnancies conceived naturally and through ART in a tertiary hospital. **Methods**: This was a retrospective cohort study of all MCDA twin pregnancies that received obstetric care and delivered at La Fe University and Polytechnic Hospital between 2015 and 2021. MCDA pregnancies that were referred to the tertiary hospital for specialized management, follow-up, and delivery were also included. The study was approved by The Health Research Institute Hospital La Fe (IIS La Fe). **Results**: Among the 184 MCDA pregnancies, 149 (81%) had a natural conception, and 35 (19%) were conceived through ART. Patients with an MCDA pregnancy who conceived through ART had a significantly older maternal age (38.0 [35.5–42.5] vs. 32.0 [29.0–36.0], *p <* 0.001) and an elevated rate of nulliparity (80.0% vs. 50.3%, *p* = 0.001). Regarding pregnancy complications, MCDA pregnancies through ART were associated with a significantly higher incidence of gestational diabetes (22.9% vs. 2.7%, *p <* 0.001), hypertensive disorders during pregnancy (22.9% vs. 9.4%, *p* = 0.04), and other pregnancy complications such as threatened labor or preterm prelabor rupture of membranes (14.3% vs. 36.2%, *p* = 0.015), than naturally conceived MCDA pregnancies. No differences were found in the incidence of twin-to-twin transfusion syndrome (20% vs. 33.6%, *p* = 0.155). MCDA pregnancies through natural conception had a greater rate of vaginal delivery than MCDA through ART (16.8% vs. 2.9%, *p* = 0.032). When adjusted for confounding factors, MCDA pregnancies through ART were only more likely to develop gestational diabetes than those naturally conceived (aOR 7.86, 95% CI 1.55–39.87). No differences were found regarding neonatal outcomes between groups. **Conclusions**: Compared with naturally conceived MCDA twin pregnancies, those conceived through ART displayed a significantly higher risk of developing gestational diabetes. No differences regarding other pregnancy complications, mode of delivery, or neonatal outcomes were found between groups.

## 1. Introduction

Infertility is a major human reproductive health issue [1,2,3,4,5,6,7,8,9,10,11] that affects 48.5 million couples worldwide [12,13]. Infertile patients require assisted reproductive techniques (ART) such as artificial insemination (AI), in vitro fertilization (IVF), and intracytoplasmic sperm injection (ICSI) to achieve pregnancy [14,15]. Additionally, the use of ART is steadily increasing [16] given that same-sex couples [17,18,19,20,21], persons who desire to create single-parent families [18,21,22,23], and individuals who undergo fertility preservation for both medical and nonmedical reasons [24,25,26] also benefit from them to ultimately accomplish parenthood.

The incidence of twin gestations has risen over the past several decades due to advanced maternal age at conception and the increased use of ART [27]. The risk of having a dizygotic twin pregnancy increases with maternal age [28,29,30] due to the greater level of gonadotropins produced with age [29]. Indeed, the twinning rate increases by 300% between 15 and 37 years old [31]. Moreover, the more advanced the maternal age, the lower the number and quality of oocytes, and the higher the need for fertility treatment [32,33]. Twin pregnancies are associated with a higher risk of perinatal complications than singleton gestations [27,34], including preterm birth and the subsequent infant morbidity and mortality [27]. Thus, a single-embryo transfer strategy has been advocated during recent years to minimize dizygotic twin gestations [35,36,37,38,39]. Fortunately, the twin birth rate decreased by 4% during 2014–2018 [27]. Nonetheless, several studies have revealed that monozygotic twinning after single embryo transfer is more common among day 5–6 embryo transfers than among day 2–3 transfers [40,41,42,43,44,45]. Moreover, some authors have described that assisted hatching is associated with an increased risk of monozygotic twinning [42,46].

Monochorionic twin pregnancies conceived through ART have been reported to display a higher risk of second-trimester miscarriage [47], adverse perinatal outcomes [48], cesarean delivery [49], neonatal morbidity [50], and neonatal death [49,50] than those naturally conceived. However, other authors have not found differences regarding adverse perinatal outcomes [51], gestational age at delivery, rate of preterm birth, type of delivery, or admission to the neonatal intensive care unit (NICU) between monochorionic diamniotic (MCDA) twins conceived through ART and natural conception [52]. Thus, this study aimed to compare the perinatal outcomes of MCDA twin pregnancies conceived naturally and through ART in a tertiary hospital.

## 2. Materials and Methods

This was a retrospective cohort study among all MCDA twin pregnancies that received obstetric care and delivered at La Fe University and Polytechnic Hospital, Valencia, Spain. MCDA pregnancies that were referred to the tertiary hospital for specialized management, follow up, and delivery were also included. All MCDA twin pregnancies from June 2015 to December 2021 were included. Data of the included patients were collected from the digital clinical history of the hospital. The study was approved by The Health Research Institute Hospital La Fe (IIS La Fe).

Gathered maternal information included age, body mass index, nulliparity, and smoking habit. Considered pregnancy outcomes were miscarriage, considered as pregnancy loss before 24 weeks of gestation; gestational diabetes; hypertensive disorders of pregnancy that included chronic hypertension, gestational hypertension, pre-eclampsia, eclampsia, and HELLP syndrome; twin-to-twin transfusion syndrome; selective fetal growth restriction; fetal growth restriction of both twins; other pregnancy complications that included cholestasis, gestational hypothyroidism, short cervix, cervical insufficiency, threatened preterm labor, previable preterm premature rupture of membranes, and preterm premature rupture of membranes; and mode of delivery. The neonatal outcomes that were retrieved from the first and second newborn at birth involved birth weight, Apgar score, pH of the artery and vein of the umbilical cord, NICU admission, demise, morbidity during the first 30 days of life, and chronic neonatal morbidity.

R version 4.0.3 (The R Foundation for Statistical Computing) was used for the statistical analysis. Quantitative data are shown as mean and interquartile range, while categorical data are presented as absolute and relative frequencies. Comparisons between the characteristics of the groups were performed using Student’s *t*-test or Kruskal–Wallis test for continuous variables, and Fisher’s exact testing for categorical variables. Multivariable logistic regression analyses were used, and the adjusted odds ratio (aOR) and the 95% confidence interval (CI) are reported. The odds ratios were adjusted by maternal age, nulliparity, body mass index, and smoking habit.

## 3. Results

A total of 184 MCDA twin pregnancies were included. Among them, 149 (81%) were natural conception, and 35 (19%) were conceived through ART (Table 1).

Patients with an MCDA twin pregnancy who conceived by ART displayed a significantly higher maternal age than those with an MCDA gestation naturally conceived (38.0 (35.5–42.5) vs. 32.0 (29.0–36.0), *p <* 0.001). Additionally, women with an MCDA gestation through ART had an elevated rate of nulliparity compared with those with a naturally conceived MCDA twin pregnancy (80.0% vs. 50.3%, *p* = 0.001, Table 1).

Regarding pregnancy complications, MCDA twin pregnancies conceived through ART were associated with a significantly higher incidence of gestational diabetes (22.9% vs. 2.7%, *p <* 0.001), hypertensive disorders during pregnancy (22.9% vs. 9.4%, *p* = 0.04), and other pregnancy complications such as threatened preterm labor or preterm prelabor rupture of membranes (14.3% vs. 36.2%, *p* = 0.015) than in naturally conceived MCDA pregnancies (Table 2). Interestingly, no differences were found in the incidence of twin-to-twin transfusion syndrome between MCDA twin pregnancies conceived through ART and those naturally conceived (20% vs. 33.6%, *p* = 0.155).

The mode of delivery was compared between MCDA twin pregnancies naturally conceived and those through ART (Table 2). Naturally conceived MCDA twin pregnancies showed a greater rate of vaginal delivery than MCDA through ART (16.8% vs. 2.9%, *p* = 0.032).

**Figure 1:**
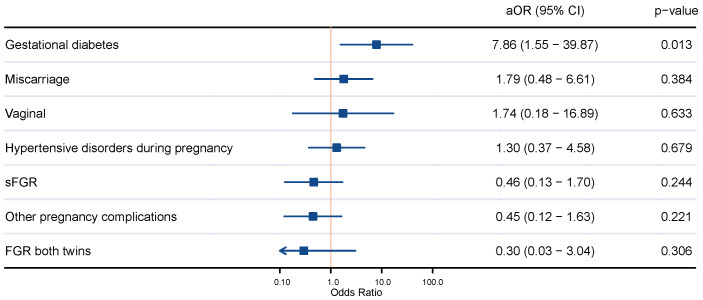
Comparison of pregnancy outcomes of naturally conceived monochorionic diamniotic twin pregnancies and those through assisted reproductive techniques. Adjusted odds ratio (aOR) by maternal age, nulliparity, body mass index, and smoking habit; 95% confidence interval (CI); vaginal delivery (vaginal); selective fetal growth restriction (sFGR); fetal growth restriction (FGR).

Multivariate analysis revealed that MCDA twin pregnancies through ART were more likely to develop gestational diabetes than those naturally conceived (aOR 7.86, 95% CI 1.55–39.87). When adjusting for confounding factors including maternal age, nulliparity, body mass index, and smoking habit, no statistical differences were found regarding other pregnancy complications or mode of delivery between MCDA twin pregnancies naturally conceived and those through ART (Figure 1).

Neonatal outcomes including birthweight, Apgar score, NICU admission, and neonatal morbidity were compared between MCDA twin gestations that were naturally conceived and those through ART. Nonetheless, no differences were found regarding neonatal outcomes (Table 3). When comparing specific neonatal outcomes of the first and the second newborn at delivery between MCDA twin pregnancies naturally conceived and those through ART, no differences were found between the groups (Table 4).

## 4. Discussion

The main findings of this study are that, compared with naturally conceived MCDA twin pregnancies, those conceived through ART have a significantly higher incidence of gestational diabetes. Remarkably, no differences regarding other pregnancy complications, mode of delivery, or neonatal outcomes were found.

The occurrence of twin gestations has risen worldwide over the recent decades due to advanced maternal age at conception and the heightened use of ART [27]. Actually, the twinning rate rose by 76% from 1980 to 2009 (19 to 33 twins per 1000 births), was stable from 2009 to 2012, and rose until 2014 (34 per 1000 births), before declining by 8% from 2014 to 2020 [53]. Noticeably, from 2020 to 2021, the twinning rates were the lowest in two decades (31 per 1000 births) [53]. Twin pregnancies are associated with a higher risk of perinatal mortality and morbidity than singleton gestations [27,34]. Thus, a single-embryo transfer strategy has been advocated during recent years to lessen dizygotic twin gestations [35,36,37,38,39]. Nevertheless, it has been reported that ART increases the incidence of monozygotic twins from 1 in 250 in natural conceptions to approximately 1 in 50 [41,47]. Particularly, extended culture or embryo transfer among days 5–6 [40,41,42,43,44,45] and assisted hatching [42,46] have been described to confer a higher risk of monozygotic twinning after single-embryo transfer. Due to vascular anastomoses [54], monochorionic twin pregnancies are associated with higher perinatal morbidity and mortality than dichorionic twin gestations [50,55]. Transfusion imbalances through the vascular anastomoses cause pregnancy complications specific to MCDA twin pregnancies [54,56]. In this regard, 10% of MCDA twin pregnancies develop twin–twin transfusion syndrome (TTTS), and 5% develop twin anemia polycythemia sequence (TAPS) [54,56].

ART increases the rate of monochorionic twin pregnancies [41,47], and MCDA gestations are at a particularly elevated risk of adverse outcomes due to placental vascular anastomoses [54,56]. Thus, the present study assessed whether perinatal outcomes are more adverse in MCDA twin pregnancies conceived through ART than in those naturally conceived. Not surprisingly, patients with an MCDA pregnancy who conceived through ART displayed a significantly higher maternal age and an elevated rate of nulliparity. Accordingly, Simoes et al. compared MCDA twins conceived naturally and through ART and revealed that women pregnant through ART had a significantly more advanced maternal age and were more often nulliparous [48].

Concerning pregnancy complications, the present study shows that MCDA twin pregnancies through ART were associated with significantly higher incidences of gestational diabetes, hypertensive disorders during pregnancy, and other pregnancy complications such as threatened preterm labor or preterm prelabor rupture of membranes than naturally conceived MCDA twin pregnancies. Nonetheless, when adjusting for confounding factors, MCDA twin pregnancies conceived via ART were only more likely to develop gestational diabetes than naturally conceived MCDA twin pregnancies. These results are in line with those in the available literature. Prats et al. performed a retrospective cohort study and revealed that MCDA twin pregnancies conceived through ART had a heightened risk of gestational diabetes than naturally conceived MCDA twin gestations [52]. No differences were found between groups regarding gestational age at delivery, onset of labor, preterm birth, or intrauterine growth restriction [52]. Similarly, a recent meta-analysis did not find significant differences regarding hypertensive disorders of pregnancy, very preterm delivery, risk of intrauterine death, and small for gestational age fetuses between MCDA twin pregnancies conceived naturally and through ART [49]. Identically, a study conducted by Tronjer-Bregar et al. concluded that MCDA twins conceived through ART were not associated with an increased risk of adverse perinatal outcomes compared with spontaneous MCDA twins [51]. Nevertheless, other authors have reported that MCDA twin pregnancies conceived through ART display more adverse perinatal outcomes than naturally conceived MCDA pregnancies [47,57]. Couck et al. carried out a retrospective cohort study of MCDA twin pregnancies conceived after ART or naturally and concluded that MCDA twins by ART displayed reduced survival rates and larger rates of second-trimester miscarriage than the naturally conceived MCDA twins [47]. Similarly, Sun et al. conducted a retrospective review and described an increased risk of preterm premature rupture of membranes in MCDA twin pregnancies conceived via ART compared with in those naturally conceived [57]. Hence, it remains controversial whether the mode of conception of MCDA twin pregnancies has a negative impact on pregnancy complications. However, the updated evidence reveals that MCDA twin pregnancies conceived via ART only have a higher risk of gestational diabetes and not of other pregnancy complications compared with naturally conceived MCDA twin pregnancies.

Noticeably, no differences were found in the incidence of twin-to-twin transfusion syndrome between naturally conceived MCDA twin pregnancies and those conceived through ART in the present study. Accordingly, both the retrospective cohort study of Couck et al. and the meta-analysis of Wang et al. have revealed that the mode of conception of MCDA twin pregnancies had no impact on the risk of TTTS [47,49]. Thus, the available evidence shows no differences in the incidence of TTTS between MCDA twin pregnancies conceived naturally and through ART.

Regarding the mode of delivery, naturally conceived MCDA pregnancies in the present study showed a greater rate of vaginal delivery than MCDA conceived through ART. Nonetheless, when adjusted by confounding factors including maternal age, nulliparity, body mass index, and smoking habit, no statistical differences were found regarding the mode of delivery between naturally conceived MCDA twin pregnancies and those through ART. Similarly, neither Couck et al. nor Prats et al. found differences with respect to the incidence of cesarean delivery between MCDA twin pregnancies conceived after ART or naturally [47,52]. Nonetheless, a meta-analysis regarding monochorionic twin pregnancies conceived by ART vs. naturally revealed that monochorionic twin pregnancies conceived through ART display a higher risk of cesarean section [49]. Hence, when adjusted by confounding factors, the mode of conception in MCDA twin pregnancies does not appear to affect the mode of delivery.

Importantly, no differences regarding neonatal outcomes were found in the present study between MCDA twin pregnancies conceived naturally and through ART. Similarly, Couck et al. and Prats et al. did not find differences regarding weight discordance [47,52], birth weight [47], and admission to the NICU [52] between MCDA twin pregnancies conceived after ART or naturally. Nevertheless, Simoes et al. revealed that monochorionic twins conceived via ART had a lower mean birth weight than those naturally conceived [48]. Additionally, the meta-analysis by Wang et al. and a retrospective cohort study by Hack et al. have described that MCDA twin pregnancies conceived via ART displayed a heightened risk of neonatal deaths compared to those naturally conceived [49,50]. Therefore, it is still unclear whether the mode of conception of MCDA twin pregnancies has a negative impact on neonatal outcomes.

The primary importance of this study is that it adds to the scientific evidence regarding the perinatal outcomes of MCDA twin pregnancies according to the mode of conception. Drawbacks of the present work include the limited sample size. Further studies should be carried out in order to clarify whether the mode of conception of MCDA twin pregnancies has an impact on perinatal outcomes.

In conclusion, the present study shows that, when adjusting for confounding factors, MCDA twin pregnancies conceived through ART have a significantly higher incidence of gestational diabetes than naturally conceived MCDA twin gestations. Noticeably, no differences regarding other pregnancy complications, mode of delivery, and neonatal outcomes were found between naturally conceived MCDA twin pregnancies and those through ART. These findings are reassuring for both healthcare professionals and patients. Nonetheless, additional studies are required to confirm these findings and to appropriately counsel pregnant women with MCDA twin pregnancies.

## Figures and Tables

**Table 1:** Characteristics of pregnant women with a monochorionic diamniotic twin pregnancy according to the mode of conception.

	Total Sample (n = 184)	ART (n = 35)	Natural Conception (n = 149)	*p*
Maternal age	33.0 (30.0–37.0)	38.0 (35.5–42.5)	32.0 (29.0–36.0)	<0.001
Nulliparity	103 (56.0)	28 (80.0)	75 (50.3)	0.001
BMI	25.0 (22.7–28.3)	27.0 (22.7–30.2)	25.0 (22.7–28.0)	0.103
Smoking habit	21 (11.4)	2 (5.7)	19 (12.8)	0.376

Assisted reproductive techniques (ART); body mass index (BMI).

**Table 2:** Pregnancy outcomes of monochorionic diamniotic twin pregnancies through natural conception vs. through assisted reproductive techniques.

Pregnancy Complications	Total Sample (n = 184)	ART (n = 35)	Natural Conception (n = 149)	*p*
Hypertensive disorders during pregnancy	22 (12.0)	8 (22.9)	14 (9.4)	0.04
Other pregnancy complications	59 (32.1)	5 (14.3)	54 (36.2)	0.015
Miscarriage	24 (13.4)	5 (14.3)	19 (13.2)	0.789
TTTS	57 (31.0)	7 (20.0)	50 (33.6)	0.155
FGR both twins	7 (3.8)	1 (2.9)	6 (4.0)	1.0
sFGR	39 (21.2)	5 (14.3)	34 (22.8)	0.359
Gestational diabetes	12 (6.5)	8 (22.9)	4 (2.7)	<0.001
Vaginal delivery	26 (14.1)	1 (2.9)	25 (16.8)	0.032

Assisted reproductive techniques (ART); hypertensive disorders of pregnancy included chronic hypertension, gestational hypertension, pre-eclampsia, eclampsia, or HELLP syndrome; other pregnancy complications included cholestasis, gestational hypothyroidism, short cervix, cervical insufficiency, threatened preterm labor, previable preterm premature rupture of membranes, and preterm premature rupture of membranes; miscarriage was considered as pregnancy loss before 24 weeks of gestation; twin-to-twin transfusion syndrome (TTTS); fetal growth restriction (FGR); selective fetal growth restriction (sFGR).

**Table 3:** Neonatal outcomes of monochorionic diamniotic twin pregnancies naturally conceived vs. through assisted reproductive techniques.

Neonatal Outcomes	Total Sample (n = 368)	ART (n = 70)	Natural Conception (n = 298)	*p*
Birthweight (grams)	2082.5 (1485.0–2420.0)	2110.0 (1466.3–2453.8)	2075.0 (1500.0–2395.0)	0.634
Male sex	148 (50.3)	20 (35.7)	128 (53.8)	0.017
Apgar score 1	9.0 (8.0–9.0)	9.0 (7.0–9.0)	9.0 (8.0–9.0)	0.107
Apgar score 5	10.0 (9.0–10.0)	10.0 (9.0–10.0)	10.0 (9.0–10.0)	0.797
Apgar score 10	10.0 (10.0–10.0)	10.0 (10.0–10.0)	10.0 (10.0–10.0)	0.963
pHa	7.300 (7.260–7.330)	7.280 (7.250–7.320)	7.310 (7.268–7.333)	0.015
pHv	7.340 (7.310–7.370)	7.330 (7.290–7.360)	7.350 (7.310–7.370)	0.061
NICU admission	94 (33.6)	19 (33.9)	75 (33.5)	1.0
Demise first 30 days	14 (4.9)	1 (1.8)	13 (5.7)	0.317
Demise after 30 days	4 (1.5)	0 (0.0)	4 (1.8)	0.586
Neonatal morbidity first 30 days	116 (41.3)	23 (41.1)	93 (41.3)	1.0
Chronic neonatal morbidity	43 (16.0)	6 (10.9)	37 (17.3)	0.306

Assisted reproductive techniques (ART); neonatal intensive care unit (NICU).

**Table 4:** Neonatal outcomes of the first and the second newborn at delivery of monochorionic diamniotic twin pregnancies through natural conception vs. through assisted reproductive techniques.

Neonatal Outcomes	Total Sample (n = 184)	ART (n = 35)	Natural Conception (n = 149)	*p*
Birthweight newborn 1 (grams)	2080.0 (1580.0–2427.5)	2245.0 (1593.8–2480.0)	2080.0 (1580.0–2420.0)	0.692
Male sex newborn 1	78 (50.3)	11 (36.7)	67 (53.6)	0.108
Apgar score 1, newborn 1	9.0 (8.0–9.0)	9.0 (7.3–9.0)	9.0 (8.0–9.0)	0.708
Apgar score 5, newborn 1	10.0 (9.0–10.0)	10.0 (9.0–10.0)	10.0 (9.0–10.0)	0.513
Apgar score 10, newborn 1	10.0 (10.0–10.0)	10.0 (10.0–10.0)	10.0 (10.0–10.0)	0.584
pHa, newborn 1	7.3 (7.3–7.3)	7.3 (7.3–7.3)	7.3 (7.3–7.3)	0.22
pHv, newborn 1	7.4 (7.3–7.4)	7.4 (7.3–7.4)	7.4 (7.3–7.4)	0.549
NICU admission, newborn 1	51 (34.7)	12 (40.0)	39 (33.3)	0.523
Demise newborn 1 first 30 days	8 (5.3)	0 (0.0)	8 (6.7)	0.358
Demise newborn 1 after 30 days	2 (1.4)	0 (0.0)	2 (1.8)	1.0
Neonatal morbidity newborn 1 first 30 days	61 (41.5)	13 (43.3)	48 (41.0)	0.838
Chronic neonatal morbidity, newborn 1	21 (14.9)	3 (10.0)	18 (16.2)	0.566
Birthweight newborn 2 (grams)	2085.0 (1450.0–2380.0)	2105.0 (1451.3–2413.8)	2055.0 (1450.0–2375.0)	0.869
Male sex newborn 2	70 (50.4)	9 (34.6)	61 (54.0)	0.085
Apgar score 1, newborn 2	9.0 (8.0–9.0)	9.0 (7.3–9.0)	9.0 (8.0–9.0)	0.052
Apgar score 5, newborn 2	10.0 (9.0–10.0)	10.0 (9.0–10.0)	10.0 (9.0–10.0)	0.744
Apgar score 10, newborn 2	10.0 (10.0–10.0)	10.0 (10.0–10.0)	10.0 (10.0–10.0)	0.383
Days of hospital admission, newborn 2	7.0 (3.0–24.0)	5.5 (3.0–19.8)	7.0 (3.0–24.0)	0.698
Demise newborn 2 first 30 days	6 (4.4)	1 (3.8)	5 (4.6)	1.0
Demise newborn 2 after 30 days	2 (1.5)	0 (0.0)	2 (1.9)	1.0
Neonatal morbidity newborn 2 first 30 days	55 (41.0)	10 (38.5)	45 (41.7)	0.827
pHa, newborn 2	7.3 (7.3–7.3)	7.3 (7.2–7.3)	7.3 (7.3–7.3)	0.018
pHv, newborn 2	7.3 (7.3–7.4)	7.3 (7.3–7.4)	7.3 (7.3–7.4)	0.032
NICU admission, newborn 2	43 (32.3)	7 (26.9)	36 (33.6)	0.642
Chronic neonatal morbidity, newborn 2	22 (17.2)	3 (12.0)	19 (18.4)	0.564

Assisted reproductive techniques (ART); neonatal intensive care unit (NICU).

## Data Availability

The data in this study were obtained from the clinical program of the University and Polytechnic Hospital La Fe. Such a dataset may be completely available on request to the corresponding author. The data are not publicly available due to privacy.
